# Historicizing Indian Psychiatry

**DOI:** 10.4103/0019-5545.31568

**Published:** 2006

**Authors:** Debashis Chatterjee

**Affiliations:** * Practising Psychiatrist, P-199, Block-A, Bangur Avenue, Calcutta 700055, West Bengal; e-mail: d.chatterjee@vsnl.net

Sir,

Amit Ranjan Basu's article ‘Historicizing Indian Psychiatry’^1^ and the commentaries on it by Paul Hoff^2^ and Sridhar Sharma^3^ invoke some critical comments.

Dr Sharma completely misses the point of ‘historicism’ (as noted by A.R. Basu himself) and provides an ‘account/appraisal’ of Indian history that has little relevance to the issue. Or may be, in an ironical sense, this brings forth the importance of Foucault's methodology in understanding/managing our past. Even as a simple historical story telling, the article contains some gross inaccuracies and sweeping generalizations that need critical reflection.

1. The battle of Buxar in 1764 was not ‘between the East India Company under the leadership of Lord Clive and Nawab Sirajjudaula’. Sirajjudaula was defeated in 1757 battle, apprehended within a few days by the company sepoys and was murdered by Mohammedi Beig at the instigation of the company. The 1764 battle was between the company on one hand and Mir Kasim, Shah Alam II and nawab of Audh on the other. Neither was Clive the leader of the company forces at that time. He was in England though he came back soon afterwards at the helm of the company.
2. Neither Charaka nor Hippocrates were individuals in history but represent schools of thought and systems of medicine. Hippocratic times were 600 to 300 BC. The Hippocratic oath that we know of today was formulated sometime in 3rd century BC. Ayurvedic times are taken as 8th century BC to 8th century AD. The Charaka Samhita (a compilation of the then thoughts and practices) is a product of 1st century AD. It is definitely post-Alexander. Thus the claim that Indian humor theory predates Greek one is completely untenable. The glaring fact that is being ignored is that at that time there was a fair bit of business and cultural exchange between west and east and all ideas, be it about medicine or astronomy or mathematics evolved in an environment of exchange.
3. ‘Philosophy in India is essentially spiritual’—this often quoted sentiment is itself a post-colonial construction that arose from the need to create an east-west dichotomy. As a strategy of recovery of pre-colonial self it is flawed and is historically completely incorrect.
4. Dr Sharma mentions, in a general way, a multitude of psychotherapeutic theories and practices mentioned in ancient Indian text, many in as yet untranslated Sanskrit texts. It will be nice to have definitive examples. To represent a glorified and sanskritized one-dimensional version of past in this subcontinent is a crypto-political move that has been going on for too long. Invoking and interpreting Gita and some such other texts in exclusivity to construct our psychiatric past by other authors reflect the same phenomenon. Psychiatry is a product of post-industrial paradigm and there can be an (ancient) history of psychiatry but there cannot be an ancient psychiatry.

Both Paul Hoff and Sharma tend to equate Foucault with antipsychiatry. This reflects a rather poor reading of Foucault and antipsychiatry both. Given that Laing latched on to Foucault's work, Szasz was rather non-committal. Both of them attacked the then psychiatry from an Existential point but Szasz's was a Judeo-Christian moralistic over interpretation of Sartre while Laing was notorious for continuously shifting his theoretical positions and practices. On the other hand Cooper was a member of South African Communist Party and his criticisms were made from that angle. Foucault himself had little to say about antipsychiatry (or psychiatry for that matter) and his study of madness and unreason was an example to demonstrate a larger project of unraveling historical development of ‘ideas and practices’.

In Basu's writing ‘Indian Psychiatry’ and ‘psychiatry in India’ are used interchangeably and without any specificity. Does he consider the two to be equivalent? I think that will be rather naïve and anti-Foucauldian in itself. Basu is also, rightly, concerned about the construction of *lack* resulting from the power play that goes behind the development of episteme in a Foucauldian sense but does not distinguish it from the power play that generates in colonial exchange that precludes dialogue and fosters silence. He is also rather vague in pointing out how to address this *lack* and listen to it in a turn around of Foucauldian method. That is a bit disappointing. In trying to be too Foucauldian, Basu also ignores Derrida's criticisms of Foucault's reading of Cartesian reason–unreason.
